# A retrospective analysis of normal saline and lactated ringers as resuscitation fluid in sepsis

**DOI:** 10.3389/fmed.2023.1071741

**Published:** 2023-04-06

**Authors:** Shahin Isha, Parthkumar H. Satashia, Siva Naga S. Yarrarapu, Austin B. Govero, Michael F. Harrison, Hassan Z. Baig, Pramod Guru, Anirban Bhattacharyya, Colleen T. Ball, Sean M. Caples, Ami A. Grek, Michael R. Vizzini, Syed Anjum Khan, Katherine J. Heise, Hiroshi Sekiguchi, Warren L. Cantrell, Jeffrey D. Smith, Sanjay Chaudhary, Karthik Gnanapandithan, Kristine M. Thompson, Charles G. Graham, Jed C. Cowdell, Aleksandra Murawska Baptista, Claudia R. Libertin, Pablo Moreno Franco, Devang K. Sanghavi

**Affiliations:** ^1^Department of Critical Care Medicine, Mayo Clinic, Jacksonville, FL, United States; ^2^Department of Emergency Medicine, Mayo Clinic, Jacksonville, FL, United States; ^3^Department of Quantitative Health Sciences, Mayo Clinic, Jacksonville, FL, United States; ^4^Division of Pulmonary and Critical Care Medicine, Mayo Clinic, Rochester, MN, United States; ^5^Department of Critical Care Medicine, Mayo Clinic Health System Mankato, Mankato, MN, United States; ^6^Department of Critical Care Medicine, Mayo Clinic, Phoenix, AZ, United States; ^7^Division of Hospital Internal Medicine, Mayo Clinic, Jacksonville, FL, United States; ^8^Division of Infectious Diseases, Mayo Clinic, Jacksonville, FL, United States

**Keywords:** sepsis, fluid dose, balanced solution, ringers lactate, normal saline, resuscitation

## Abstract

**Background:**

The Surviving Sepsis Campaign suggested preferential resuscitation with balanced crystalloids, such as Lactated Ringer’s (LR), although the level of recommendation was weak, and the quality of evidence was low. Past studies reported an association of unbalanced solutions, such as normal saline (NS), with increased AKI risks, metabolic acidosis, and prolonged ICU stay, although some of the findings are conflicting. We have compared the outcomes with the preferential use of normal saline vs. ringer’s lactate in a cohort of sepsis patients.

**Method:**

We performed a retrospective cohort analysis of patients visiting the ED of 19 different Mayo Clinic sites between August 2018 to November 2020 with sepsis and receiving at least 30 mL/kg fluid in the first 6 h. Patients were divided into two cohorts based on the type of resuscitation fluid (LR vs. NS) and propensity-matching was done based on clinical characteristics as well as fluid amount (with 5 ml/kg). Single variable logistic regression (categorical outcomes) and Cox proportional hazards regression models were used to compare the primary and secondary outcomes between the 2 groups.

**Results:**

Out of 2022 patients meeting our inclusion criteria; 1,428 (70.6%) received NS, and 594 (29.4%) received LR as the predominant fluid (>30 mL/kg). Patients receiving predominantly NS were more likely to be male and older in age. The LR cohort had a higher BMI, lactate level and incidence of septic shock. Propensity-matched analysis did not show a difference in 30-day and in-hospital mortality rate, mechanical ventilation, oxygen therapy, or CRRT requirement. We did observe longer hospital LOS in the LR group (median 5 vs. 4 days, *p* = 0.047 and higher requirement for ICU post-admission (OR: 0.70; 95% CI: 0.51–0.96; *p* = 0.026) in the NS group. However, these did not remain statistically significant after adjustment for multiple testing.

**Conclusion:**

In our matched cohort, we did not show any statistically significant difference in mortality rates, hospital LOS, ICU admission after diagnosis, mechanical ventilation, oxygen therapy and RRT between sepsis patients receiving lactated ringers and normal saline as predominant resuscitation fluid. Further large-scale prospective studies are needed to solidify the current guidelines on the use of balanced crystalloids.

## 1. Introduction

The Third International Consensus Guidelines defined sepsis as a “life-threatening organ dysfunction caused by a dysregulated host immune response to infection.” On the other hand, septic shock has been defined as “a vasopressor requirement to maintain a mean arterial pressure of 65 mmHg or greater and serum lactate level greater than 2 mmol/L in the absence of hypovolemia” in a patient with suspected or confirmed sepsis (1). Sepsis and septic shock as a disease entity confer a major burden on the healthcare system and rigorous attempts have been made to improve the overall mortality and morbidity by adjusting the guidelines as per existing evidence (2, 3).

Early diagnosis and initiation treatment, which comprises of antibiotics and judicious fluid therapy during the initial phase of resuscitation have received strong recommendations. The 2021 updates on International Guidelines for Management of Sepsis and Septic Shock strongly recommended the use of crystalloids as first-line fluid therapy during the initial resuscitation phase of sepsis or septic shock. Moreover, for adults with sepsis or septic shock, the use of balanced crystalloids over normal saline was suggested by the guideline although the quality of evidence was reported to be “low” and the recommendation was “weak” (4).

Balanced crystalloid solutions, such as lactated Ringer’s solution (LR), Ringer Acetate, Plasmalyte, etc., are usually normotonic and have a lower tendency to cause hyperchloremic acidosis (5, 6). On the other hand, the normal saline (NS) solution is an unbalanced solution and has been associated with hyperchloremic metabolic acidosis (7, 8). A large volume of NS infusion may also cause coagulopathy, renal dysfunction, and impaired immunological response (9). Despite significant work that has been done on the use of balanced crystalloids in critically ill patients, the data regarding the crystalloid of choice in sepsis is conflicting.

A large retrospective cohort study done by Raghunathan et al. (10) on patients admitted with sepsis failed to demonstrate any difference in the incidence of AKI and in-hospital and ICU length of stay, although the mortality rates were lower in the balanced crystalloid cohort. The SMART trial also showed favorable outcomes (lower rates of mortality, renal replacement therapy, or persistent renal dysfunction) in critically ill patients treated with balanced crystalloid use compared to normal saline (11). A network meta-analysis done by Rochwerg et al. (12) revealed that fluid resuscitation with balanced crystalloids was associated with lower mortality compared to normal saline in patients with sepsis, based on an indirect comparison. On the other hand, a chloride-restrictive strategy during fluid resuscitation of critically ill patients was shown to have a lower incidence of acute kidney injury (AKI) and renal replacement therapy requirement in a sequential prospective study conducted by Yunos et al. (13) however, no difference in mortality, hospital stay, or ICU stay was noted.

In the background of conflicting evidence, we have tried to compare the outcomes associated with NS and LR as a resuscitation fluid in patients who presented to the emergency department and were subsequently diagnosed with sepsis.

## 2. Materials and methods

Our automated data pull identified 2,899 hospitalization in 2,751 sepsis patients who presented to the Emergency Departments of 19 different Mayo Clinic sites between August 2018 to November 2020 with a diagnosis of sepsis and were treated with ≥30 mL/kg of either NS or LR during the first 6 h. Patients were excluded (*n* = 221) if they declined research authorization, were under 18 years old at the time of presentation to the ED or if the date of diagnosis was missing. Patients were also excluded (*n* = 571) they received >30 ml/kg of both fluid type or, received <30 ml/kg of each fluid type during the first 6 h. If a patient had more than one hospitalization with a diagnosis of sepsis during the study period, then we selected the first encounter per patient for inclusion in the study. Our final analysis included 2022 unique patients of these, 1,428 (70.6%) received NS, and 594 (29.4%) received LR as the predominant fluid. Patient information and relevant data were collected from the Electronic Health record. The time of diagnosis was determined by either time of antibiotic administration or the time of a lactic acid draw, not the result. Primary outcomes include in-hospital death, and secondary outcomes include in-hospital length-of-stay, 30-day mortality, ICU admission after diagnosis, ventilator use, and CRRT use. We used mean arterial pressure (MAP), vasopressor requirement and lactate level to define septic shock retrospectively. Any patient with a vasopressor requirement to maintain a mean arterial pressure of 65 mm Hg or greater and serum lactate level greater than 2 mmol/L was identified to have septic shock, as per the “Third International Consensus Definitions for Sepsis and Septic Shock” guidelines (1).

Mayo Clinic Institutional Review Board (IRB) granted an exemption (application number 20-008691) from the need for approval for our study on 3 September 2020. The need for informed consent was waived by our IRB. Procedures were followed in accordance with the ethical standards of the committee responsible for human experimentation and with the Helsinki Declaration of 1975.

### 2.1. Statistical analysis

Continuous characteristics were summarized with the sample median and interquartile range. Categorical characteristics were summarized with the frequency and percentage of patients. Characteristics known at the time of sepsis diagnosis were summarized according to fluid type among the cohort of patients who received 30 ml/kg or more of fluid. We aimed to estimate the effect of fluid type on outcomes (in-hospital mortality (primary), hospital LOS (secondary), death within 30 days of diagnosis (secondary), ICU admission after diagnosis (secondary), mechanical ventilator (secondary), adult oxygen therapy (secondary), and CRRT (secondary). To control confounding, propensity score matching was used to identify a cohort of patients with similar baseline characteristics. Propensity score is defined here as the conditional probability of a patient diagnosed with sepsis receiving predominantly lactated ringers vs. normal saline given a set of covariates known at the time of sepsis diagnosis (baseline).

A logistic regression model with fluid type as the dependent variable and all the baseline characteristics displayed in Table 1A as covariates was used to estimate the propensity score. Due to missingness, MAP was categorized based on tertiles and missing values were assigned to as separate category to estimate the propensity score. The nearest neighbor matching algorithm was used to select one patient who received normal saline to each patient who received lactated ringers with a caliper width equal to 0.2 of the standard deviation (SD) of the logit of the propensity score. The matching algorithm additionally included body mass index with a caliper width of 5 kg/m^2^ and total fluid amount with a caliper width of 5 ml/kg. Standardized differences were estimated before and after matching using the tableone R package to assess potential imbalance in baseline characteristics between the 2 groups (14). A standardized difference less than 0.10 for a given baseline characteristic was considered a negligible imbalance between groups (substantial imbalance was defined as a standardize difference >0.2). Odds ratios (OR) and Hazard ratios (HR) were estimated from single variable logistic regression (categorical outcomes) and Cox proportional hazards regression (hospital length of stay). For hospital LOS, censoring occurred at the date of death for those who had an inpatient death and the Kaplan-Meier method was used to estimate median (25th percentile, 75th percentile); an HR less than 1.00 indicates a worse outcome (longer length of stay) for patients who were given LR compared to patients who were given NS. For categorical outcomes, ORs greater than 1.00 indicate a worse outcome for patients who were given >30 ml/kg of LR compared to patients who were given >30 ml/kg of NS. All statistical tests were two-sided. For our primary outcome, *p* > 0.05 was considered statistically significant without adjustment for multiple testing. For our secondary outcomes, *p* < 0.05 was considered statistically significant after adjustment for multiple testing using the Holm method, however, we do note unadjusted p-values in the results and tables. Adjusted p-values will be labeled as such. Statistical analyses were performed using R version 4.0.3 (R Foundation for Statistical Computing, Vienna, Austria).

**TABLE 1A:** Baseline characteristics before and after matching according to fluid type among those who received 30 ml/kg or more.

	Before matching			After matching		
	Normal saline (*N* = 1,428)	Lactated ringers (*N* = 594)	SMD	Normal saline (*N* = 436)	Lactated ringers (*N* = 436)	SMD
Age at diagnosis (years)	71 (60, 81)	68 (58, 79)	0.11	69 (59, 79)	69 (59, 80)	0.04
Male sex	640 (44.8%)	238 (40.1%)	0.10	170 (39.0%)	188 (43.1%)	0.08
Race			0.13			0.06
American Indian/Alaskan Native	10 (0.7%)	3 (0.5%)		4 (0.9%)	3 (0.7%)	
Asian	32 (2.2%)	14 (2.4%)		11 (2.5%)	12 (2.8%)	
Black	33 (2.3%)	22 (3.7%)		13 (3.0%)	15 (3.4%)	
Native Hawaiian/Pacific Islander	3 (0.2%)	2 (0.3%)		1 (0.2%)	1 (0.2%)	
White	1,312 (91.9%)	528 (88.9%)		390 (89.4%)	392 (89.9%)	
Other/unknown	38 (2.7%)	25 (4.2%)		17 (3.9%)	13 (3.0%)	
Ethnicity			0.08			0.03
Not Hispanic or Latino	1,353 (94.7%)	554 (93.3%)		411 (94.3%)	409 (93.8%)	
Hispanic or Latino	41 (2.9%)	18 (3.0%)		14 (3.2%)	14 (3.2%)	
Other/Unknown	34 (2.4%)	22 (3.7%)		11 (2.5%)	13 (3.0%)	
Body mass index	24.6 (21.5, 28.7)	28.7 (23.9, 34.9)	0.60	26.2 (23.0, 30.6)	26.8 (23.2, 31.5)	0.02
COPD	173 (12.1%)	62 (10.4%)	0.05	40 (9.2%)	43 (9.9%)	0.02
Hypertension	530 (37.1%)	225 (37.9%)	0.02	171 (39.2%)	161 (36.9%)	0.05
CKD	236 (16.5%)	103 (17.3%)	0.02	74 (17.0%)	75 (17.2%)	0.01
Diabetes	276 (19.3%)	101 (17.0%)	0.06	85 (19.5%)	71 (16.3%)	0.08
CAD	254 (17.8%)	88 (14.8%)	0.08	69 (15.8%)	73 (16.7%)	0.03
CHF	154 (10.8%)	62 (10.4%)	0.01	47 (10.8%)	41 (9.4%)	0.05
Obesity	231 (16.2%)	184 (31.0%)	0.35	100 (22.9%)	107 (24.5%)	0.04
Dialysis	48 (3.4%)	19 (3.2%)	0.01	11 (2.5%)	13 (3.0%)	0.03
Type of diagnosis: lactate-draw	806 (56.4%)	434 (73.1%)	0.35	311 (71.3%)	304 (69.7%)	0.04
Hospital type (destination)	834 (58.4%)	469 (79.0%)	0.45	338 (77.5%)	344 (78.9%)	0.03
MAP	90.2 (81.3, 99.3)	88.7 (79.0, 98.3)	0.14	89.0 (80.0, 99.1)	88.7 (79.3, 98.3)	0.03
Septic shock	95 (6.7%)	113 (19.0%)	0.38	64 (14.7%)	67 (15.4%)	0.02
Blood culture positive	94 (6.6%)	58 (9.8%)	0.12	41 (9.4%)	44 (10.1%)	0.04
Maximum lactate	3.3 (2.6, 4.6)	3.8 (2.9, 5.7)	0.30	3.9 (2.9, 5.5)	3.7 (2.9, 5.8)	0.07
Total fluid amount (ml/kg)	40.3 (33.8, 50.9)	49.6 (39.9, 63.0)	0.04	44.7 (37.2, 55.2)	45.4 (37.7, 55.9)	0.05

SMD, standardized mean difference; COPD, chronic obstructive pulmonary disease; CKD, chronic kidney disease; CAD, coronary artery disease; CHF, congestive heart failure; MAP, mean arterial pressure. Numerical characteristics are given as median (interquartile range), while categorical characteristics are given as the percentage of patients. Overall maximum lactate was not available 740 patients before matching and 252 patients after matching.

## 3. Results

**FIGURE 1:**
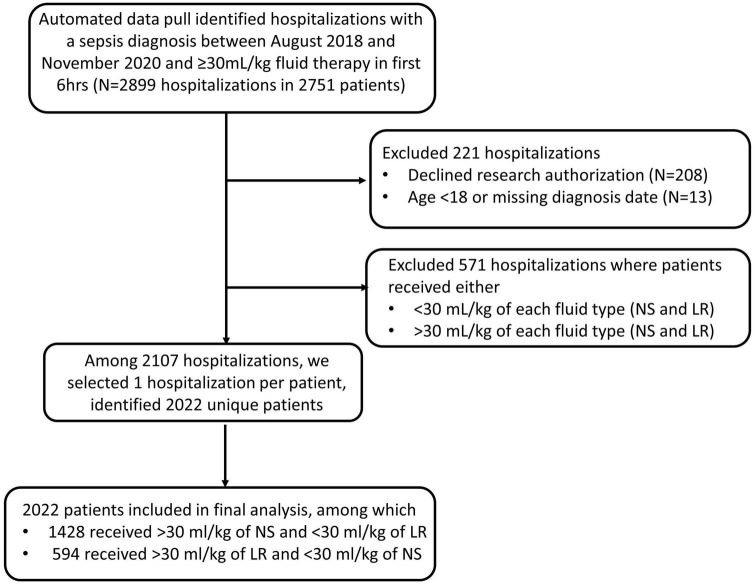
Flowchart showing inclusion and exclusion of patients in the study cohort.

Our retrospective analysis included 2022 patients; of these, 1,428 (70.6%) received NS, and 594 (29.4%) received LR as the predominant fluid (Figure 1). Baseline patient characteristics are summarized in Table 1A before and after propensity score matching. Before matching, there were substantial baseline differences (standardized difference >0.2) between LR and NS, where patients who received predominantly LR were more likely to have had a higher body mass index and/or history of obesity, a lactate draw diagnosis, treated at a destination hospital, had septic shock, and a higher overall maximum lactate. After matching, all standardized differences were 0.08 or less and considered negligible. Table 1B shows baseline vital signs and laboratory information according to fluid type after matching. We did not observe any substantial imbalance in baseline vital signs and labs after matching (all standardized differences ≤0.18).

**TABLE 1B:** Baseline vital signs and laboratory information of RL and NS cohorts after matching.

	Normal saline > 30 ml/kg (*N* = 436)	Lactated ringers > 30 ml/kg (*N* = 436)	SMD
Temperature (F)	*n* = 436	*n* = 435	
	98.1 (97.5, 98.4)	98.1 (97.5, 98.4)	0.13
Systolic blood pressure (mmHg)	*n* = 436	*n* = 436	
	123 (110, 138)	123 (111, 137)	0.02
Diastolic blood pressure (mmHg)	*n* = 436	*n* = 436	
	71 (62, 80)	71 (61, 80)	0.06
Pulse (beats per minute)	*n* = 435	*n* = 435	
	82 (71, 94)	81 (71, 92)	0.06
Respirations (breaths per minute)	*n* = 431	*n* = 427	
	18 (16, 20)	18 (16, 19)	0.03
Oxygen saturation (%)	*n* = 65	*n* = 61	
	95.1 (92.6, 97.0)	95.2 (92.8, 97.4)	0.08
Hemoglobin (g/dL)	*n* = 436	*n* = 435	
	11.9 (10.0, 13.7)	11.4 (9.6, 12.9)	0.18
White blood cell count (x10^3^/mcL)	*n* = 436	*n* = 435	
	13.4 (8.4, 18.2)	12.4 (7.2, 17.4)	0.07
Platelets (x10^3^/mcL)	*n* = 436	*n* = 435	
	208 (139, 280)	213 (149, 282)	0.06
Sodium P (mmol/L)	*n* = 301	*n* = 294	
	135 (132, 139)	136 (133, 139)	0.15
Potassium P (mmol/L)	*n* = 299	*n* = 295	
	4.0 (3.7, 4.5)	4.1 (3.7, 4.5)	0.02
BUN P (mg/dL)	*n* = 301	*n* = 293	
	22 (15, 33)	25 (16, 39)	0.18
Creatinine P (mg/dL)	*n* = 301	*n* = 293	
	1.2 (0.8, 1.6)	1.2 (0.8, 1.9)	0.11
Lactate P (mmol/L)	*n* = 320	*n* = 321	
	2.3 (1.6, 3.7)	2.1 (1.4, 3.2)	0.14
Bicarbonate P (mmol/L)	*n* = 301	*n* = 294	
	22 (19, 25)	22 (19, 24)	0.06

SMD, standardized difference. The number of available observations and median (25th percentile, 75th percentile) are shown.

Table 2 summarizes the outcomes according to the fluid category. In our matched cohort, 8.0% died in-hospital among those who predominantly received >30 mL/kg of NS, and 8.5% died in-hospital among the group who predominantly received >30 mL/kg of LR. We did not find evidence of an association of fluid type (LR vs. NS) with in-hospital mortality (OR 1.06; 95% CI 0.66–1.73; *p* = 0.81) (primary outcome). The median hospital LOS after diagnosis was 1 day longer for those who received LR vs. NS (5 vs. 4 days; *p* = 0.046) (secondary outcome). In the subset of patients not in the ICU at the time of diagnosis, we found those who received LR were less likely to be admitted to the ICU after diagnosis compared to those who received NS (36.5% vs. 45.2%; OR 0.70; 95% CI 0.51–0.96; *p* = 0.026) (secondary outcome). We did not find evidence of differences in 30-day mortality or requirements for mechanical ventilator, adult oxygen therapy, or CRRT (all *p* ≥ 0.066). After adjustment for multiple testing, associations of fluid type with hospital LOS and ICU admission after diagnosis were no longer statistically significant (adjusted *p* = 0.28 and 0.18, respectively).

**TABLE 2:** Association of fluid type with outcomes in the matched LR and NS cohorts.

Outcome	Normal saline (*N* = 436)^a^	Lactated ringers (*N* = 436)^a^	OR or HR (95% CI), LR vs. NS^b^	Unadjusted *p*-value
**Primary outcome**				
In-hospital mortality	35 (8.0%)	37 (8.5%)	1.06 (0.66–1.73)	0.81
**Secondary outcomes**				
Hospital LOS	4 (3, 7)	5 (3, 8)	0.87 (0.76–1.00)	**0.047**
Death within 30 days of diagnosis	69 (15.8%)	76 (17.4%)	1.12 (0.79–1.61)	0.52
ICU admission after diagnosis^c^	159/352 (45.2%)	107/293 (36.5%)	0.70 (0.51–0.96)	**0.026**
Mechanical ventilator	39 (8.9%)	56 (12.8%)	1.50 (0.98–2.32)	0.066
Adult oxygen therapy	187/326 (57.4%)	178/323 (55.1%)	0.91 (0.67–1.24)	0.56
CRRT	13/326 (4.0%)	12/323 (3.7%)	0.93 (0.41–2.08)	0.86

*P*-value of 0.05 was considered to be cut-off for statistical significance. Values shown in bold font represent statistical significance.

OR, odds ratio; HR, hazard ratio; CI, confidence interval; LOS, length of stay.

^a^Number (percent) or sample median (25th percentile, 75th percentile). Median hospital LOS was estimated using the Kaplan-Meier method censoring at the day of death for those patients who died in-hospital.

^b^ORs and HRs were estimated from single variable logistic regression (categorical outcomes) and Cox proportional hazards regression (hospital length of stay). For hospital length of stay, censoring occurred at the date of death for those who had an inpatient death and an HR less than 1.00 indicates a worse outcome (longer length of stay) for patients who were given LR compared to patients who were given NS. For categorical outcomes, ORs greater than 1.00 indicate a worse outcome for patients who were given >30 ml/kg of LR compared to patients who were given >30 ml/kg of NS.

^c^In subset of patients not admitted to the ICU prior to diagnosis.

## 4. Discussion

In our original cohort, we noted that most of the patients (1,428/2,022) were predominantly treated with >30 mL/kg of NS. Those treated with LR tended to have a higher BMI and were sicker with 19.0% having septic shock vs. 6.7% among those who received NS. Prior to matching patients on baseline characteristics and fluid amount, we observed in-patient mortality rates of 10.9% among those who received LR and 6.4% among those who received NS. After matching, in-patient mortality rates were similar (8.5 and 8.0%, respectively). For our secondary outcomes, after matching we observed a slight increase in hospital LOS and lower rate of ICU admission after diagnosis among those who had LR vs. NS, but these would not be considered statistically significant after adjustment for multiple testing based on 8 hypothesis tests of secondary outcomes. Such study findings contrast with some of the prior studies that reported favorable outcomes with balanced crystalloids (10, 13, 15).

While most of these studies were limited by their retrospective design, findings from the recent clinical trials have tried to shed more light on this domain. The SPLIT multicenter, double-blinded, cluster randomized, double-crossover clinical trial on 2,278 eligible medical and surgical ICU patients did not demonstrate any difference in AKI-related outcomes and mortality rate between buffered crystalloid and normal saline as fluid therapy (16). Although the SPLIT trial did not focus on sepsis patients, a similar finding was reported by the SALT pilot trial comprising of 974 patients with septic patients comprising 25 and 28% of balanced crystalloid and normal saline groups, respectively, in line with the findings of our study (17). On the other hand, the SMART single-center, randomized trial reported a lower rates of 30-day inpatient mortality and adverse renal outcome with the use of balanced crystalloid in their subgroup analysis of sepsis patients (18).

Limitations related to patient demographics and site-specific variations in outcome may be present in some of these past literatures. In that aspect, this study has several strengths. With more than 2,000 patients from 19 sites, both academic and community, from across multiple regions, including the Southeast, Southwest, and Midwestern United States adds to the generalizability. We have large sample size in our original cohort and even our sample size after matching would have at least 80% power at the two-sided 5% significance level to detect a difference of at least 6% in in-patient mortality (for example, 5% vs. 10%, 10% vs. 16%). Considering the current evidence on varying patient outcomes based on culture-status and fluid overload, our propensity-matched analysis addresses potential confounders and strengthens the overall methodology (19, 20).

The is a retrospective analysis, which comes with inherent limitations. We did not stratify our cohort based on the site of infection and origin of sepsis, which can obscure some of the findings. Also, there was no standardization of rate, type, or mode of delivery of fluid resuscitation among our patients, all of which could have a significant impact on the outcome. Particularly, the lack of information on infusion rate is a salient limitation, as previous studies have shown better survival with quicker rates (21, 22). Additionally, we could not account for prehospital fluid administration and partial administration of different fluid types leading to potential mixed effects. Finally, our study cohort had more than 90% of white patients, which is non-representative of the overall US population, although the collection of data from 19 different sites provides generalizability. With the contrasting pieces of data, a further large-scale study is needed on the outcome of different types of resuscitation fluid in sepsis.

## 5. Conclusion

In our study, we did not show any difference in outcomes with LR as a predominant fluid for sepsis resuscitation compared to NS. Additional evidence is warranted to solidify the current guidelines on the use of balanced crystalloids.

## Data availability statement

The original contributions presented in this study are included in the article/supplementary material, further inquiries can be directed to the corresponding author.

## Ethics statement

Mayo Clinic Institutional Review Board (IRB) granted an exemption (application number 20-116 008691) from the need for approval for our study. The need for informed consent was waived by our IRB. The study was conducted in accordance with national and international guidelines.

## Author contributions

SI: conceptualization, writing–original draft, writing–review and editing, visualization, supervision, and project administration. PS: conceptualization, writing–original draft, writing–review and editing, investigation, and visualization. SY: conceptualization, writing–review and editing, visualization, and data curation. AG and MH: methodology, writing–review and editing, visualization, investigation, and data curation. HB, PG, and AB: conceptualization, writing–original draft, writing–review and editing, visualization, and supervision. CB: methodology, writing–review and editing, data curation, and formal analysis. SMC, AG, MV, SK, KH, HS, WC, JS, SC, KG, KT, CG, JC, and AM: conceptualization, writing–original draft, and writing–review and editing. CL and PM: conceptualization, writing–original draft, writing–review and editing, and supervision. DS: conceptualization, writing–original draft, writing–review and editing, visualization, supervision, and project administration. All authors contributed to the article and approved the submitted version.
